# Vibration Response Signal Analysis of Gear Transmission System Considering the Influence of Coupled Crack Fault

**DOI:** 10.3390/s26051615

**Published:** 2026-03-04

**Authors:** Hengzhe Shi, Wei Li, Wanlin Zhou

**Affiliations:** 1 College of Mechanical and Electrical Engineering, Nanjing University of Aeronautics and Astronautics, Nanjing 210000, China; shihengzhe@nuaa.edu.cn; 2 School of Mechanical Engineering, University of Science and Technology Beijing, 30 Xueyuan Road, Haidian District, Beijing 100083, China; liwei@me.ustb.edu.cn

**Keywords:** fault signal detection, gear system dynamic response, dynamics model, root crack, time-varying meshing stiffness

## Abstract

Accurate fault diagnosis of gear transmission systems is crucial for ensuring mechanical reliability and preventing catastrophic failures. However, existing research predominantly focuses on single-gear crack faults, often overlooking the complex coupling effects when cracks occur simultaneously on meshing gears in practical engineering scenarios. To address this research gap, a multi-degree-of-freedom dynamic model incorporating time-varying mesh stiffness under normal, single-crack, and coupled-crack conditions is established. Experimental validation is conducted based on an FZG closed test rig for power flow. The results indicate that the mesh stiffness under coupled-crack conditions is generally lower than that under single-crack conditions. In the time-domain vibration response, the periodic impact amplitudes induced by coupled cracks are significantly amplified, with the impact period jointly influenced by the rotational speeds of both the driving and driven gears. According to frequency-domain analysis, coupled cracks result in a notable increase in harmonic peaks of the mesh frequency, enhanced sideband amplitudes, and a modulation period that is between the rotational frequencies of the driving and driven gears. The simulation results from the dynamic model show high consistency with the experimental signals in terms of time-frequency characteristic trends and time-domain indicators such as the crest factor, thereby validating the effectiveness of the dynamic model. This study elucidates the unique influence mechanism of coupled cracks on the dynamic behavior of gear systems and can provide theoretical guidance for the accurate diagnosis and condition assessment of multi-tooth faults in subsequent research.

## 1. Introduction

Gears, as the core components of mechanical transmission systems, suffer from tooth root crack faults that are one of the primary causes of abnormal system vibration, noise, and even catastrophic failure. The initiation and propagation of cracks alter the system’s dynamic characteristics by reducing the time-varying mesh stiffness (TVMS), thereby affecting load distribution, vibration response, and fatigue life. Therefore, in-depth research on crack evolution mechanisms and fault characteristics is of significant importance for achieving accurate condition monitoring and life prediction.

Regarding crack modeling and stiffness calculation, early studies often relied on simplified assumptions, making it difficult to accurately characterize stiffness degradation behavior under large cracks. Chaari et al. [1] derived an analytical stiffness formula for cracked gears and validated it via finite element analysis; Ma et al. [2] further considered the root circle-base circle offset and precise transition curves, proposing three methods for calculating crack stiffness. They confirmed that assuming the limit line as a parabola could significantly improve calculation accuracy under large crack conditions; Mohammed et al. [3] developed a new method capable of accurately calculating stiffness for cracks up to 50% of the tooth root thickness, effectively enhancing the accuracy of dynamic response simulations. For helical gear systems, Wang et al. [4] proposed an improved model that comprehensively considers tooth, transverse, and axial foundation stiffnesses, revealing the differential stiffness degradation patterns of two crack types: tooth tip propagation and end face propagation; Yang et al. [5] distinguished between crack locations at the tooth root and tooth flank, addressing the previous research gap of insufficient attention to tooth flank cracks. Furthermore, Ma et al. [6] considered the extended tooth-contact effect caused by gear flexibility, revealing a coupling mechanism in which cracks degrade the gear body stiffness, thereby indirectly weakening the stiffness of healthy teeth. Notably, traditional crack propagation simulations often neglect the dynamic coupling effect of “crack-dynamic load,” which may lead to distorted path predictions. Yu et al. [7] established an integrated finite-element-dynamics iterative model, achieving high-fidelity propagation path simulation that considers fillet crack excitation and dynamic load feedback; Ouyang et al. [8] proposed an improved model coupling tooth-foundation stiffness degradation and, combined with residual signal processing and multiple statistical indicators, achieved quantitative characterization of crack evolution patterns. Additionally, Xiao et al. [9] constructed a friction dynamics model for composite cracks (straight cracks evolving into arc/parabolic shapes) under mixed lubrication conditions based on fractal theory. Their work confirmed that crack geometry has a dominant influence on mesh stiffness degradation, providing a new perspective for crack morphology identification based on dynamic responses.

In the field of dynamic modeling, researchers have progressively evolved from simplified models to high-fidelity system-level models. Wu et al. [10] analyzed the influence of cracks on the vibration response of a single-stage gearbox based on a lumped parameter model and compared the sensitivity of various statistical indicators; Omar et al. [11] developed a nine-degree-of-freedom model coupling gearbox structure and shaft vibration, incorporating time-varying mesh stiffness and transmission error displacement excitation, achieving good agreement between simulation and experiment under multiple operating conditions; Wan et al. [12] established a lateral-torsional coupled dynamic model, revealing the periodic impulse response induced by cracks, the increase in impact amplitude, and the amplitude modulation characteristics caused by geometric transmission errors. For the complex structure of planetary gear sets, Wang et al. [13] systematically revealed the differential influence patterns of cracks at different locations (sun gear, planet gears, and ring gear) on load-sharing characteristics through an 18-degree-of-freedom model; Liu et al. [14] and Han et al. [15], based on loaded tooth contact analysis and fracture mechanics theory, respectively, constructed improved models considering actual crack paths and time-varying transducer distances, finding that ring gear cracks have a weaker impact on load distribution among external meshing pairs; Duan et al. [16] validated theoretical crack morphology through crack propagation experiments, revealing the coupled influence of initial crack location, ring gear boundary conditions (pin support/outer rim constraint), and housing flexibility on fault characteristics, noting that increased housing flexibility, while reducing vibration amplitude, can diminish fault identifiability. In multi-stage transmission systems, Qiao et al. [17] developed a two-stage cracked gear dynamic model considering actual crack propagation paths, revealing a cross-stage fault propagation mechanism whereby a single-stage crack can induce rotational sideband features in a healthy stage through coupling effects.

In terms of fault diagnosis methods, researchers have focused on extracting weak fault features from strong noise and non-stationary interference. Cai and Li [18] utilized the Generalized S-transform to construct time-frequency filtering factors for adaptive denoising, effectively suppressing the masking effect of strong noise on tooth profile crack features in mining machinery; Merainani et al. [19] proposed the HEWT method, which integrates Empirical Wavelet Transform and Hilbert Transform, overcoming the mode mixing and end effects of HHT and demonstrating higher sensitivity to early-stage root crack damage in noisy environments; Teng et al. [20] developed a sparse representation method based on Tunable Q-factor Wavelet Transform, combined with normalized multi-level envelope spectrograms, achieving accurate extraction of weak compound fault features in low-speed planetary gear sets. Regarding feature indicators, Ma and Chen [21] systematically revealed the essential differences between crack and spalling faults by combining nonlinear dynamics methods such as phase trajectories and fractal dimensions; Jia and Howard [22] found that amplitude and phase modulation features in coherent time synchronous averaging signals can effectively distinguish between localized spalling and crack damage; Ma et al. [23] further proposed two new indicators with high sensitivity in both time and frequency domains, enabling effective identification of different crack propagation stages. Yang et al. [24] developed an analytical model for time-varying excitation induced by surface defects in rolling element bearings, demonstrating that precise mechanism modeling is fundamental for generating discriminative fault features. Niola et al. [25] applied advanced accelerometric signal processing to monitor the torque/speed equilibrium point in aircraft hybrid electric propulsion systems, highlighting the practical value of combining physical insights with signal analytics for real-time condition monitoring.

Previous research has predominantly focused on analyzing scenarios where cracks appear on a single gear, examining the meshing stiffness, dynamic characteristics, and other parameters under healthy and faulty conditions. However, there has been relatively little investigation into cases where crack faults occur simultaneously on both gears of a meshing pair. In this paper, the condition where cracks appear simultaneously on both gears of a gear pair is defined as the coupled crack state. Specifically, the ‘coupled crack’ state is explicitly defined herein as the scenario where both the driving and driven gears in a meshing pair simultaneously exhibit root crack faults, leading to interactive stiffness degradation. This definition distinguishes our work from traditional single-gear fault studies. The gear transmission system is studied comparatively under three conditions: normal state, single crack state, and coupled crack state. Despite these advancements, current technical states predominantly rely on single-fault assumptions, neglecting the nonlinear interaction effects between simultaneous faults. This limitation hinders the accurate prediction of vibration characteristics in complex failure scenarios. To address these limitations, the main contributions of this work are summarized as follows:

(1) A time-varying mesh stiffness model considering the coupling effect of simultaneous cracks on meshing gears is established, revealing the superimposed degradation mechanism.
(2) The unique vibration response characteristics of coupled cracks are elucidated through both simulation and experiment, specifically identifying the modulation period variation lying between driving and driven rotational frequencies.
(3) The proposed dynamic model is validated via an FZG test rig, providing a reliable theoretical basis for multi-tooth fault diagnosis in engineering applications.

The results verify that the vibration responses obtained from the established dynamic model are consistent with the trends of the signals extracted from the experiments, with only minor discrepancies. Understanding these coupled mechanisms is critical for developing robust condition monitoring systems in industrial applications, such as wind turbine gearboxes and automotive transmissions, where multi-tooth faults are common. This study aims to bridge the gap between theoretical modeling and practical engineering monitoring needs, offering potential strategies for early warning of complex gear failures.

## 2. Time-Varying Meshing Stiffness Considering Crack Coupling Effect

### 2.1. Time-Varying Meshing Stiffness Model of Gear Transmission System

**Figure 1:**
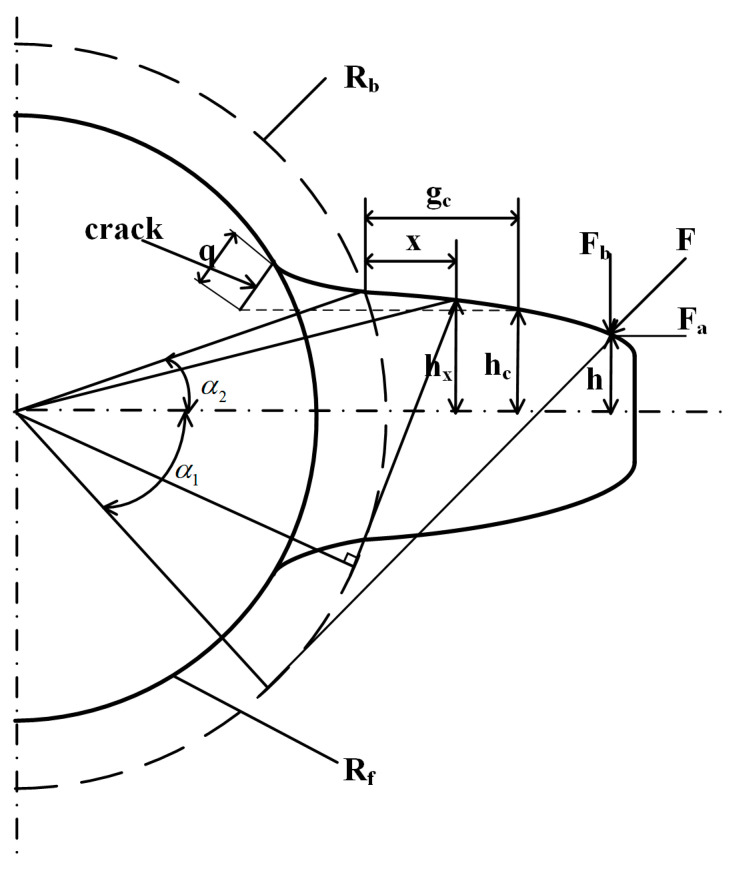
Calculation of time-varying meshing stiffness by the energy method in a cracked tooth.

The time-varying meshing stiffness is a crucial parameter in the dynamic modeling of gear transmission systems, with root crack faults predominantly affecting this parameter. Based on the potential energy method, stiffness calculation models under various conditions are established, as illustrated in Figure 1.

In the energy method, the potential energy stored in a meshing gear is assumed to comprise four components: Hertzian energy (*U_h_*), bending energy (*U_b_*), compressive energy (*U_a_*), and shear energy (*U_s_*). These energies are individually employed to calculate the Hertzian contact stiffness (*k_h_*), bending stiffness (*k_b_*), compressive stiffness (*k_a_*), and shear stiffness (*k_s_*). The overall mesh stiffness is obtained as the series combination of these individual stiffnesses. According to the principles of elastic mechanics and mechanics of materials, the following parameters are defined:

(1) $$U_{h}=\frac{F^{2}}{2k_{h}}$$

(2) $$U_{b}=\frac{F^{2}}{2k_{b}}=\int_{0}^{d}\frac{{{(F}_{b}\left(d-x\right)-F_{a}h)}^{2}}{2EI_{x}}dx+\int_{0}^{R_{b}-R_{f}}\frac{{{(F}_{b}\left(d+x_{1}\right)-F_{a}h)}^{2}}{2EI_{x}}dx_{1}$$

(3) $$U_{a}=\frac{F^{2}}{2k_{a}}=\int_{0}^{d}\frac{1.2F_{a}^{2}}{2GA_{x}}dx+\int_{0}^{R_{b}-R_{f}}\frac{1.2F_{a}^{2}}{2GA_{x}}dx_{1}$$

(4) $$U_{s}=\frac{F^{2}}{2k_{s}}=\int_{0}^{d}\frac{1.2F_{b}^{2}}{2GA_{x}}dx+\int_{0}^{R_{b}-R_{f}}\frac{1.2F_{b}^{2}}{2GA_{x}}dx_{1}$$

*F* represents the meshing force at the contact point, oriented along the line of engagement; *F_a_* is the radial component of *F*; *F_b_* is the tangential component; *E* denotes the elastic modulus; *G* is the shear modulus; *I_x_* is the moment of inertia of the tooth section measured at a distance *x* from the base circle; *A_x_* is the cross-sectional area; *d* is the horizontal distance between the meshing point and the base circle; *h* is the distance between the meshing point and the symmetry line of the gear teeth; *x* is the distance from any point on the involute curve to the base circle; and *x*_1_ is the distance from any point on the transition curve to the root circle.

Moreover, the flexible deformation of the gear base must be taken into account. This deformation is described by the expression:

(5) $$\frac{1}{k_{f}}=\frac{{cos}^{2}\alpha }{EW}(L^{*}{\left(\frac{d}{S_{f}}\right)}^{2}+M^{*}\left(\frac{d}{S_{f}}\right)+P^{*}(1+Q^{*}{tan}^{2}\alpha ))$$

Here, *S_f_* denotes the tooth thickness at the critical section as defined by Weber, and *α* represents the pressure angle. The coefficients L*, M*, P*, and Q* are determined through polynomial fitting.

According to Equation (6), single-tooth-pair mesh stiffness can be given as:

(6) $$k=\frac{1}{\frac{1}{k_{h}}+\frac{1}{k_{b1}}+\frac{1}{k_{a1}}+\frac{1}{k_{s1}}+\frac{1}{k_{f1}}+\frac{1}{k_{b2}}+\frac{1}{k_{a2}}+\frac{1}{k_{s2}}+\frac{1}{k_{f2}}}$$

In this context, the subscripts 1 and 2 refer to the driving and driven gears, respectively.

The vertical component, *F_b_*, of the meshing force tends to propel the opening of cracks, thereby influencing the meshing stiffness of the gear teeth. When cracks occur at the root, the effective area (*A_x_*) and the moment of inertia (*I_x_*) of the tooth section, measured at a distance *x* from the gear base circle, are given by:

(7) $$A_{x}=\left\{\begin{array}{c} \left(h_{c}+h_{x}\right)L,\;\;x\leq g_{c}\\ \left(2h_{x}\right)L,\;\;x>g_{c} \end{array}\right.$$

(8) $$I_{x}=\left\{\begin{array}{c} \frac{1}{12}{\left(h_{c}+h_{x}\right)}^{3}L,\;\;x\leq g_{c}\\ \frac{1}{12}{\left(2h_{x}\right)}^{3}L,\;\;x>g_{c} \end{array}\right.$$

In these expressions, *L* represents the gear width, *h_c_* is the vertical distance from the crack tip to the gear teeth’s centerline, *h_x_* is the distance from the tooth profile at the base circle (at position *x*) to the centerline, and *g_c_* is the horizontal distance from the profile at the crack tip to the base circle. By substituting Equations (7) and (8) into Equations (1) through (6), the formulation for calculating the time-varying meshing stiffness of a spur gear with a root crack can be simplified. This formulation enables the determination of the time-varying meshing stiffness under root crack conditions, which is then applied to solve the dynamics model.

### 2.2. Stiffness Solution Results Under the Influence of Crack Coupling

**Figure 2:**
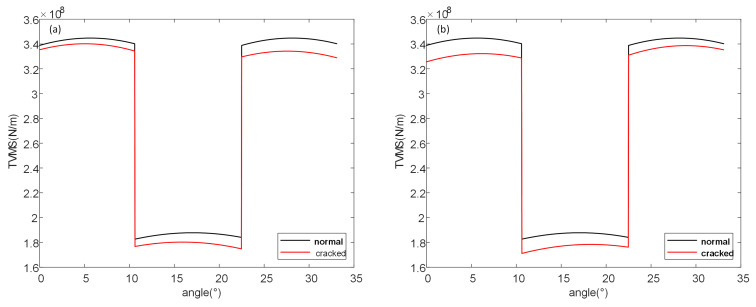
Time varying meshing stiffness of single crack failure (**a**) crack on driving gear (**b**) crack on driven gear.

Based on the aforementioned formulas and using the gear parameters provided in Table 1 (which are consistent with the experimental gear parameters in subsequent sections), the time-varying meshing stiffness over a single cycle was calculated for scenarios where cracks appear on the driving gear and the driven gear, respectively. The results are shown in Figure 2.

As can be seen in Figure 2, regardless of whether the crack appears on the driving gear or the driven gear, its stiffness decreases compared to that of the normal gear. Due to differences in meshing states, the stiffness in the double-tooth meshing zone is significantly higher than that in the single-tooth meshing zone, resulting in a distinct, abrupt change during the transition. The difference between cracks appearing on the driving gear and the driven gear is primarily reflected here. Figure 2a shows the comparison of stiffness between the cracked driving gear and the normal gear. At the abrupt transition point from the low-stiffness single-tooth meshing zone to the high-stiffness double-tooth meshing zone, the stiffness change in the cracked gear is more pronounced. When the crack is on the driving gear, the stiffness of the subsequent double-tooth meshing zone is lower than that of the preceding double-tooth meshing zone. Figure 2b shows the comparison of stiffness between the cracked driven gear and the normal gear. Unlike the case where the crack is on the driving gear, when transitioning from the high-stiffness double-tooth meshing zone to the low-stiffness single-tooth meshing zone, the stiffness change in the cracked gear is more evident. When the crack is on the driven gear, the stiffness of the subsequent double-tooth meshing zone is higher than that of the preceding double-tooth meshing zone.

**Figure 3:**
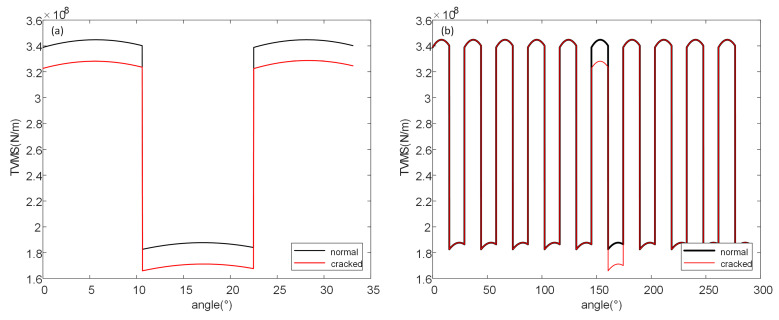
Time varying meshing stiffness of coupled crack failure (**a**) Single cycle (**b**) multiple cycles.

The differences in the two scenarios where cracks appear on the driving gear and the driven gear, respectively, were considered separately. On this basis, the coupled effect of cracks simultaneously occurring on both the driving gear and the driven gear was taken into account. The time-varying meshing stiffness of gears in both normal and cracked states was calculated for single and multiple cycles, with the results shown in Figure 3.

Evidently, when cracks are present simultaneously on both the driving gear and the driven gear, the stiffness still decreases compared to that of a normal gear pair. Comparing Figure 3 with Figure 2, when cracks appear on a pair of gears simultaneously, their influence manifests in a coupled manner, resulting in an overall stiffness that is lower than when a crack exists on a single gear. The stiffness changes at the transition points between the single-tooth and double-tooth meshing zones become more pronounced than in the case of a single cracked gear. When the gear meshing enters either the single-tooth or double-tooth meshing zone under the coupled influence of cracks on both gears, although the overall stiffness decreases more significantly in magnitude, the fluctuation trend becomes smoother.

The potential energy-based time-varying mesh stiffness model adopted in this study has been systematically validated in our previous work [26] through quantitative comparison with finite element analysis (FEA) results. The overall average stiffness values calculated by the analytical model and FEA show good agreement, with numerical errors consistently maintained within 10%. This validation confirms the accuracy and applicability of the energy method for characterizing stiffness degradation under crack faults. Therefore, the stiffness results presented in this paper, which follow the same theoretical framework, can be considered reliable for subsequent dynamic response analysis.

## 3. Vibration Response Based on the Dynamic Model of the Gear Transmission System

### 3.1. Establishment of a Dynamic Model of the Gear Transmission System

**Figure 4:**
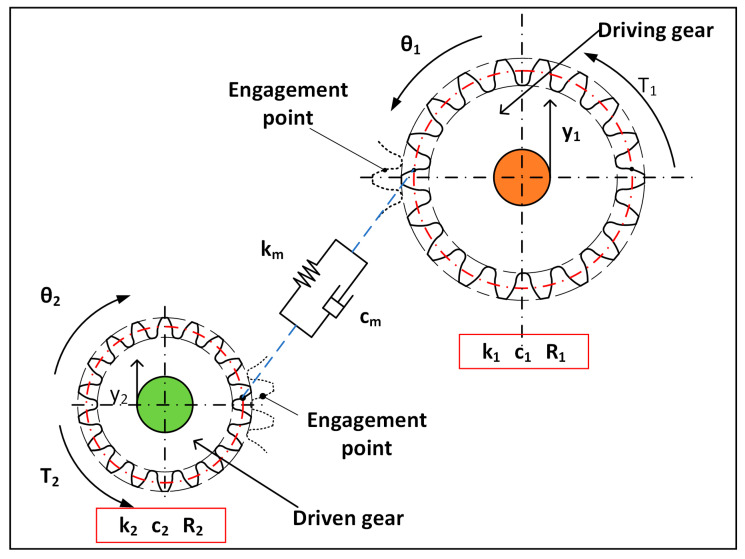
Dynamic model of spur gear pair system.

Taking into account parameters such as time-varying meshing stiffness, meshing damping, and gear transmission error, a dynamic model of the spur gear pair coupling system is established, as shown in Figure 4.

This dynamic model possesses four degrees of freedom: the rotational degrees of freedom of the driving gear and the driven gear about their respective centers of rotation, and the translational degree of freedom in the vertical y-direction. The selection of this 4-DOF configuration follows the standard lumped parameter modeling approach for spur gear pairs. It effectively captures the critical torsional vibrations of both gears and the relative translational vibration along the line of action, which are the primary contributors to meshing dynamics, while maintaining computational efficiency compared to more complex finite element models. Based on Lagrange’s general equation, the dynamic differential equations are established.

(9) $$m_{1}\ddot{y_{1}}+c_{1}\dot{y_{1}}+k_{1}y_{1}=-F_{y}$$

(10) $$I_{1}\ddot{\theta_{1}}=-T_{1}-F_{1}R_{1}$$

(11) $$m_{2}\ddot{y_{2}}+c_{2}\dot{y_{2}}+k_{2}y_{2}=F_{y}$$

(12) $$I_{2}\ddot{\theta_{2}}=-T_{2}-F_{2}R_{2}$$

(13) $$F_{y}=k_{m}\left(y_{1}+R_{1}\theta_{1}-y_{2}+R_{2}\theta_{2}-e\right)+c_{m}(\dot{y_{1}}+R_{1}\dot{\theta_{1}}-\dot{y_{2}}+R_{2}\dot{\theta_{2}}-\dot{e})$$

Regarding support stiffness and transmission error modeling, values are chosen based on the analytical method proposed by Wan et al. [12], ensuring reliable simulation of gear-rotor system dynamics. Specifically, the transmission error is modeled as a sinusoidal function of the rotational frequency, while the support damping is determined empirically. In the equation, *e* represents the geometric transmission error of the meshing gears; *m*_1_ and *m*_2_ denote the equivalent masses of the driving gear and driven gear, respectively; *I*_1_ and *I*_2_ indicate the moments of inertia of the driving gear and driven gear; *k_m_* and *c_m_* represent the meshing stiffness and meshing damping of the gear pair, respectively; *k*_1_, *k*_2_, *c*_1_, and *c*_2_ denote the support stiffness and support damping of the bearings for the driving gear and driven gear, respectively; and *R*_1_ and *R*_2_ represent the base circle radii of the driving gear and driven gear.

### 3.2. Vibration Response Analysis of Gear Transmission System

**Figure 5:**
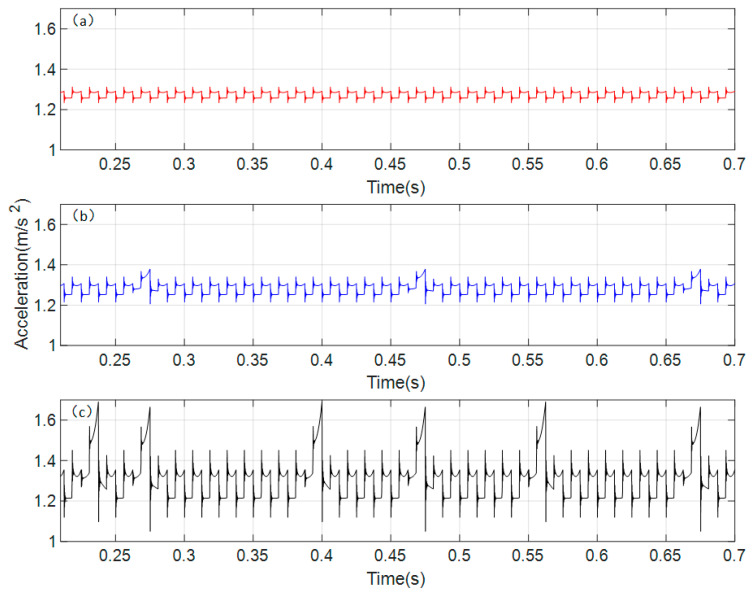
Time domain response of vibration acceleration of the spur gear pair system. (**a**) normal state (**b**) single crack state (**c**) coupled crack state.

Based on the gear parameters from Table 1 in the previous chapter, the rotational speed of the driving gear is set to 300 rpm. The dynamic model established is solved using the Runge–Kutta method, taking into account the influence of cracks under different conditions. The time-domain waveform of the vibration response obtained from the dynamic model solution is shown in Figure 5.

Figure 5a, Figure 5b, and Figure 5c, respectively, show the time-domain waveforms of the acceleration response of the spur gear pair under normal conditions, when a crack appears on a single gear, and when cracks appear simultaneously on both gears (i.e., coupled cracks). From Figure 5a, it can be observed that under normal conditions, the vibration acceleration response in the time domain is steady. Comparing with Figure 5b, the vibration acceleration response under the cracked condition exhibits periodic impacts in the time domain, and the overall vibration amplitude increases. The period of these impacts is found to be the reciprocal of the rotational frequency of the gear with the crack fault. Analyzing Figure 5c, when cracks appear simultaneously on a pair of meshing gears, the overall vibration amplitude increases significantly, and the periodic impacts are notably more pronounced compared to the single-crack condition. By comparing the amplitudes of the different periodic impacts, it is found that they correspond respectively to the rotational frequencies of the gears with crack faults. This indicates that the cracks on both faulty gears have a coupled influence on the overall time-domain response.

An analysis was conducted on the vibration acceleration time-domain response results obtained by solving the spur gear pair system under different conditions. Six time-domain indicators—maximum value, minimum value, mean value, root mean square value, peak value, and peak factor—were selected for comparison. These indicators were selected based on their distinct sensitivity to fault-induced signal characteristics. The Peak value and Peak factor are particularly effective at capturing transient impacts caused by tooth meshing irregularities, which are hallmark features of crack propagation. In contrast, Mean and RMS values reflect the overall energy levels that may remain relatively stable despite localized damage. Compared to other statistical indicators, such as Kurtosis, the Peak factor provides a normalized measure of impulsiveness that offers robustness across varying operating conditions, making it a standard and reliable choice for gear fault diagnosis. The results are presented in Table 2.

As can be seen from Table 2, from the normal state to the single-crack state and then to the coupled-crack state, the maximum value of the vibration acceleration response of the spur gear pair system in the time domain continuously increases, while the minimum value continuously decreases. Consequently, both the peak value and the peak factor continue to rise, with the differences being quite significant. Taking the peak value as an example, the peak of the time-domain response under the single-crack state can reach more than twice that of the normal state, while under the coupled-crack state, it can even exceed eight times. However, comparing the mean value and the root mean square (RMS) value reveals that the differences among the three states are not substantial, with the rate of change remaining below 3%. This indicates that when single-crack and coupled-crack faults occur, the differences in the vibration acceleration time-domain response of the spur gear pair are primarily reflected in periodic impacts, while the overall amplitude variation in the vibration response is not significant. Furthermore, regarding sensitivity to crack propagation, the comparative results suggest that Peak value and Peak factor exhibit a more pronounced monotonic increase as fault severity progresses from single to coupled conditions. This indicates they are particularly sensitive to the intensifying transient impacts associated with crack extension. In contrast, RMS values show relatively smaller variations, reflecting lower sensitivity to early-stage propagation trends. This differential sensitivity supports the selection of peak-related indicators for monitoring crack progression.

**Figure 6:**
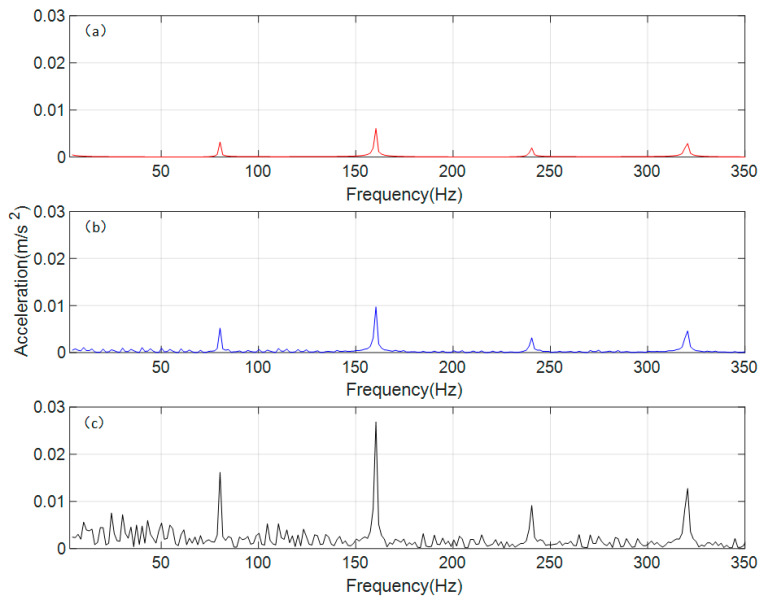
Frequency domain response of vibration acceleration of the spur gear pair system. (**a**) normal state (**b**) single crack state (**c**) coupled crack state.

The time-domain signals were processed using the Fast Fourier Transform (FFT) to obtain the vibration acceleration responses of the spur gear pair under different operating conditions in the frequency domain. The results are shown in Figure 6.

As can be seen in Figure 6, the gear meshing frequency (*f_m_*) of the gear transmission system, calculated based on relevant parameters, is 80 Hz. The differences in the frequency-domain responses under different conditions are mainly manifested at the n*f_m_* (gear meshing frequency, where *n* = 1, 2, 3, 4) positions, which correspond precisely to the peak frequencies in each interval. Under normal conditions, the frequency-domain response at n*f_m_* is relatively small, with stable fluctuations in the surrounding area. Under the single-crack condition, the amplitude at n*f_m_* increases, and fluctuating trends appear in the vicinity. Under the coupled-crack condition, the amplitude at n*f_m_* rises significantly, accompanied by distinct sideband phenomena around these frequencies. To further analyze the sideband phenomena occurring around the gear meshing frequency and its harmonics under different conditions, the vertical coordinate range in Figure 6 was adjusted from a uniform scale to an adaptive scale, with the resulting effect shown in Figure 6.

**Figure 7:**
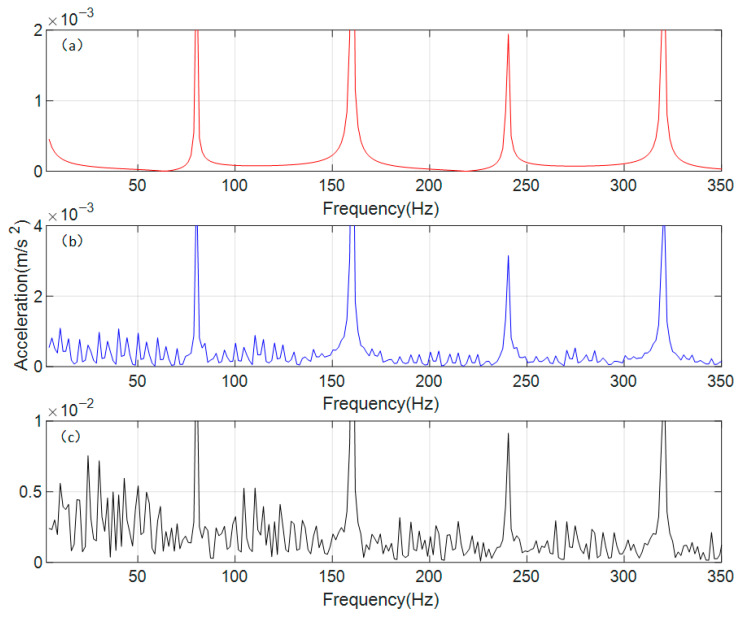
Frequency domain response of vibration acceleration of the spur gear pair system (local adaptive range). (**a**) normal state (**b**) single crack state (**c**) coupled crack state.

Figure 7a shows the gear in a normal state, which maintains a very low vibration response in all regions except the n*f_m_*. The frequency-domain response under a single-crack state is shown in Figure 7b, where lower sidebands appear around the n*f_m_*, and their period is found to be equal to the rotational frequency of the gear with the crack fault. According to Figure 7c, under the coupled-crack state, distinct sidebands emerge around the n*f_m_*; however, the regularity of their period weakens, falling between the rotational frequencies of the two gears. It occurs because cracks appear simultaneously on both gears, resulting in a coupled influence on the overall vibration response.

According to the convolution theorem, the product operation in the time domain corresponds to convolution in the frequency domain, providing a mathematical explanation for the formation mechanism of sidebands spaced at the frequency of rotation. Specifically, the time-varying stiffness induced by cracks is equivalent to a modulating signal that multiplies with the meshing-vibration carrier signal. Under single-crack conditions, the modulation frequency is singular, and the sideband spacing strictly equals the fault shaft rotational frequency. Under coupled-crack conditions, however, the modulation function incorporates dual–shaft rotational frequency components and their coupling terms, complicating the frequency-domain convolution result. This manifests as amplified sideband amplitudes and evolving spacing characteristics (interposed between the two shaft rotational frequencies). The observed vibration responses under coupled-crack conditions suggest potential nonlinear interaction effects beyond simple linear superposition. For instance, the peak factor increases by approximately eightfold under coupled cracks, which exceeds the twofold increase observed under single-crack conditions, indicating a possible nonlinear amplification mechanism. Additionally, the sideband modulation period lying between the two shaft rotational frequencies may reflect coupled modulation interactions.

## 4. Experimental Verification

### 4.1. Experimental Platform Settings

**Figure 8:**
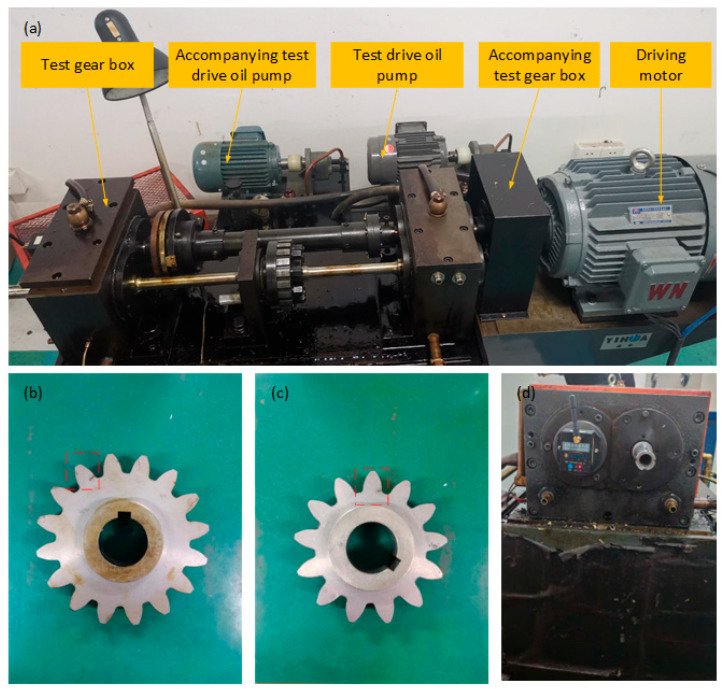
Experimental platform settings (**a**) main components of laboratory bench, (**b**) driving gear, (**c**) driven gear, (**d**) vibration acceleration sensor.

In order to validate and analyze the vibration response results obtained from the theoretical model solution, an experimental platform for a spur gear pair transmission system was constructed using an FZG closed-loop power flow test rig, as shown in Figure 8.

For the experimental signal acquisition, a wireless tri-axial acceleration sensor was employed, with the sampling frequency uniformly set to 1000 Hz. Figure 8a illustrates the main components and layout of the experimental bench, including the test gearbox, oil pump, and drive motor. Specifically, the FZG closed-loop power flow test rig adopts a back-to-back configuration, where two identical gearboxes are mechanically coupled via torque shafts to form a closed power loop. This design enables high-load testing with relatively low input power consumption, as the drive motor only needs to compensate for the frictional losses within the system. The test gearbox is lubricated by a dedicated oil pump with a controllable flow rate and temperature, ensuring consistent tribological conditions across different experimental groups. The drive motor, controlled by a variable-frequency drive, provides stable rotational speed regulation from 0 to 3000 rpm, allowing for systematic investigation of speed-dependent fault characteristics. Figure 8b,c depict the driving gear and driven gear under crack fault conditions, respectively. The gear parameters, such as module and number of teeth, are consistent with those provided in Table 1 in the previous section. The crack faults on the gears were prepared using wire cutting technology. To ensure the reproducibility and diagnostic relevance of the experimental results, the geometric parameters of the artificially introduced cracks are explicitly quantified. The crack position is at the tooth root region, with its precise location determined by the tooth root height measured from the base circle. The crack depth ratio is defined as the actual crack depth relative to the critical depth at complete tooth fracture (taken as 100%), and the experimental cracks were prepared with a depth ratio of 25%, representing an early-stage fault condition suitable for investigating initial vibration response characteristics. All cracks were fabricated using wire-electrode cutting technology with controlled machining parameters to ensure geometric consistency and repeatability across different experimental groups. This standardized preparation approach enables reliable comparison of vibration responses under normal, single-crack, and coupled-crack conditions. It is acknowledged that while this method ensures geometric consistency, it does not fully replicate the micro-structural characteristics of natural fatigue cracks. Only the gears in the fault condition are shown in the figures, while the normal condition refers to undamaged gears. In Figure 8d, the wireless vibration acceleration sensor is installed on the bearing end cover. This is because the gear-shaft-bearing-bearing end cover forms an integrated system; thus, the vibration response signal obtained from the bearing end cover corresponds to the vibration response of the gear system.

### 4.2. Experimental Result Analysis

**Figure 9:**
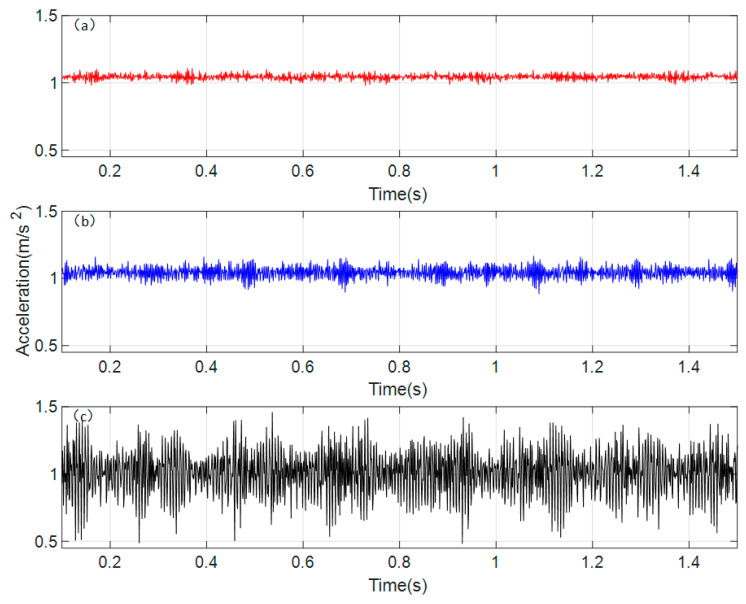
Time domain response of experimental vibration acceleration signal. (**a**) normal state (**b**) single crack state (**c**) coupled crack state.

By combining undamaged gears and gears with cracks, and replacing different gear experimental groups, the vibration acceleration responses of the spur gear transmission system under normal conditions, single-crack conditions, and coupled-crack conditions were compared. To ensure the reliability of the experimental results, each test condition was repeated three times. The qualitative trends and key vibration characteristics (e.g., periodic impacts, sideband patterns) showed good consistency across repetitions, confirming the reproducibility of the observed phenomena. The experimentally measured vibration acceleration signals were extracted to obtain the time-domain vibration acceleration responses of the gear transmission system under different conditions over a period of time, as shown in Figure 9.

In Figure 9a, the vibration acceleration signal of the spur gear pair system under normal conditions exhibits minimal fluctuation in the time domain, indicating smooth operation of the gear transmission system. In this experiment, a crack was introduced in the driving gear while the driven gear remained undamaged, simulating the vibration acceleration time-domain response of a single cracked gear in the gear pair. As shown in Figure 9b, the overall fluctuation increases significantly, showing a trend of periodic impacts. Figure 9c displays the coupled crack condition where both gears in the pair are cracked. It can be observed that the time-domain fluctuation becomes much more intense compared to the other two conditions, with clear periodic impacts appearing.

To conduct a more in-depth evaluation of the differences in the time-domain responses of the experimental gear system under different states, the vibration acceleration signal data collected from the experiments were processed based on six time-domain indicators: maximum value, minimum value, mean value, root mean square value, peak value, and peak factor. The results are presented in Table 3.

Analyzing the results in Table 3, the vibration acceleration response of the spur gear pair system shows a continuous increase in both peak values and peak factors from the normal state to the single-crack state and then to the coupled-crack state, with an accelerating rate of increase. Taking the peak value as an example, the peak of the time-domain response under the single-crack state can reach 2.2 times that under the normal state, while under the coupled-crack state, it can even approach 8 times. This is because, as the system transitions from the normal state to different crack states, the maximum value in the time domain continuously increases, the minimum value continuously decreases, and the gap between the extreme values exhibits a monotonically increasing trend. This indicates that the periodic impacts in the time domain, which emerge with the progression of crack faults, can relatively intuitively reflect the differences in the vibration acceleration responses of the experimental gear system under different states. From an overall perspective, comparing the mean and root mean square values reveals that the differences between the experimental gear system with crack faults and the normal state are not significant, with the rate of change remaining below 4%. This suggests that whether a single crack or coupled cracks are present, the overall variation in the vibration acceleration response of the experimental gear system is relatively small, making fault detection less intuitive compared to the time-domain waveform. By comparing the experimental results with those from the dynamic model in previous chapters, although the time-domain waveform of the experimental signal is less stable than that of the dynamic model, the manifested differences—namely, the periodic impacts—remain consistent. Furthermore, the discrepancies between the experimental results and the dynamic model results are minimal across different time-domain response indicators, which verifies the accuracy of the dynamic model. To quantitatively evaluate the agreement between the dynamic model predictions and experimental measurements, the percentage errors of key time-domain indicators (Mean, RMS, Peak factor, etc) were calculated under different conditions. Table 4 presents the results of percentage errors, showing that the percentage errors for all indicators across different conditions range from a minimum of 0.27% to a maximum of 6.74%, indicating that the error can be controlled within 10%. These quantitative comparisons further validate that the established dynamic model can accurately predict the vibration response characteristics of gear transmission systems under different crack fault conditions.

**Figure 10:**
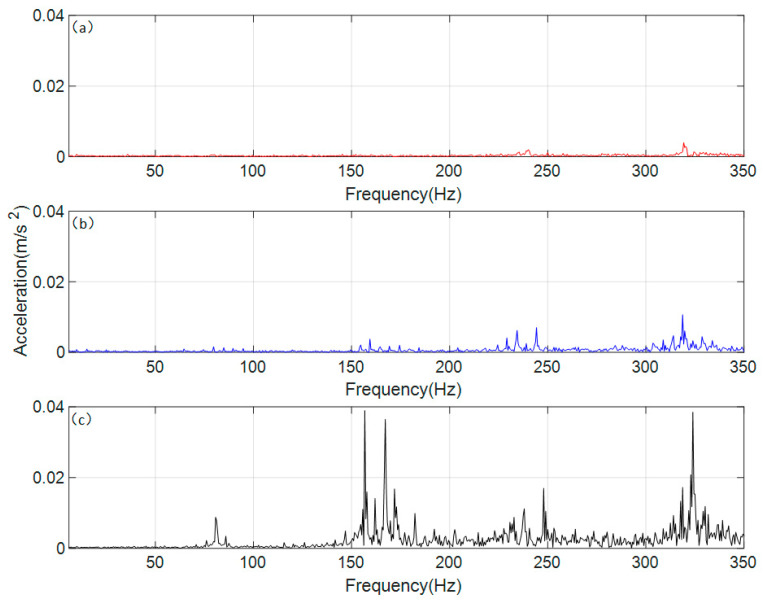
Frequency domain response of experimental vibration acceleration signal. (**a**) normal state (**b**) single crack state (**c**) coupled crack state.

Based on the parameters of the vibration acceleration sensor, a Fast Fourier Transform (FFT) model was established for the experimental signal, converting the collected vibration acceleration signal into the frequency domain, with the results shown in Figure 10.

Since the relevant experimental parameters and dynamic models remain consistent, the meshing frequency of the experimental gear transmission system is 80 Hz, still denoted as *f_m_*. To facilitate a direct comparison of peak values across different intervals, the vertical coordinate ranges in Figure 10 are kept entirely uniform. As shown in Figure 10a, the frequency-domain vibration response under normal conditions exhibits very indistinct peaks across intervals, with discernible peak frequencies only around 3*f_m_* and near 4*f_m_*, alongside a tendency for sidebands to emerge. The frequency-domain vibration response under the single-crack condition is presented in Figure 10b, where peaks and sidebands appear in the vicinity of 2*f_m_*, 3*f_m_*, and 4*f_m_*. From Figure 10c, it can be observed that under the coupled-crack condition, the peaks and sideband phenomena are highly pronounced, covering the range of n*f_m_* (*n* = 1, 2, 3, 4), and numerically far exceed those of the other two conditions.

**Figure 11:**
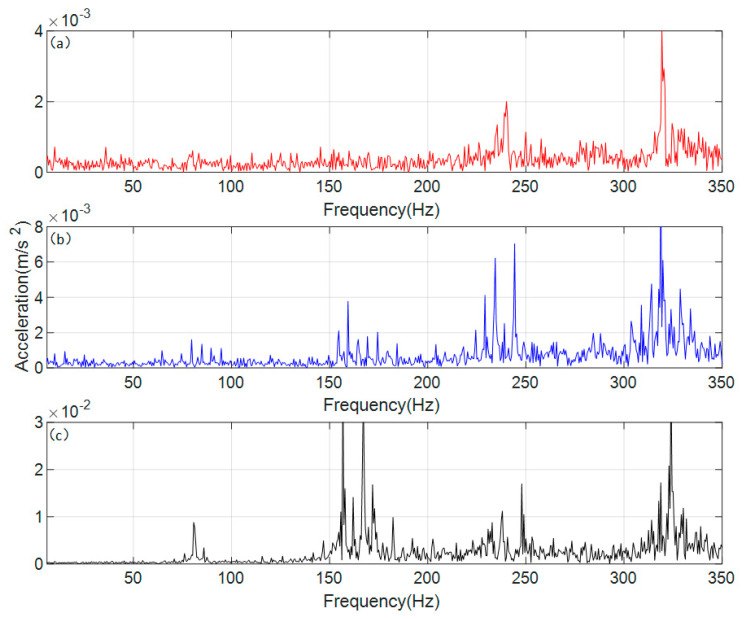
Frequency domain response of experimental vibration acceleration signal (local adaptive range). (**a**) normal state (**b**) single crack state (**c**) coupled crack state.

Since the peak values in each interval of the frequency domain under the coupled crack state shown in Figure 10c are higher compared to the other two states, the phenomena exhibited by the other two states are not sufficiently clear under a uniform vertical coordinate range. Therefore, Figure 11 is presented with an adaptive local vertical coordinate range.

Figure 11 adjusts the display range of the vertical axis to better observe and compare the characteristics of the sidebands under various conditions. In the frequency-domain response shown in Figure 11a for the normal state, periodic sidebands do not actually appear; the apparent sideband-like trend can be considered as the influence of environmental noise. In the frequency-domain response of the dynamic model under the normal state, the impact of environmental noise is simplified, so the phenomenon observed in the experimental signal does not appear. In the single-crack state shown in Figure 11b, periodic sidebands can be observed near n*f_m_* (*n* = 1, 2, 3, 4), with a period equal to the rotational frequency of the shaft where the driving gear is located. This is because the crack in the single-crack state of this experiment is set on the driving gear, which is also consistent with the frequency-domain response results of the dynamic model. In the coupled-crack state, compared with the single-crack state, the regions where periodic sidebands appear remain unchanged, but the periodicity weakens. The observed period is not equal to the rotational frequency of either the driving gear or the driven gear shaft; instead, it lies between the two rotational frequencies. A clear quantitative relationship has not yet been established, but the range of the period can be determined based on the rotational frequencies.

## 5. Discussion

Coupled crack faults induce unique dynamic behaviors distinct from single-crack scenarios. The simulation and experimental results reveal that these faults cause a superimposed degradation in mesh stiffness, highlighting a non-linear interaction at meshing transition points. In the time domain, the faults significantly amplify periodic impacts, corroborating findings on single cracks but extending them by demonstrating that coupled faults exacerbate impulsiveness due to simultaneous stiffness drops. Most notably, the frequency-domain analysis uncovers a unique sideband modulation period that differs from conventional single-crack studies. This suggests that diagnostic algorithms relying solely on single-shaft sideband spacing may miss coupled faults or misidentify the fault location. The consistency between simulation and experiment confirms that the lumped parameter model captures these coupled dynamics effectively, providing a reliable basis for future diagnostic framework development.

## 6. Conclusions

This paper establishes a dynamic model of a multi-degree-of-freedom gear transmission system, considering the influence of time-varying mesh stiffness under normal conditions, single-crack conditions, and coupled-crack conditions. The dynamic model is solved to obtain vibration acceleration response results. Based on the FZG closed power flow test rig, an experimental platform was built, and test gears in different states were prepared. The model solution results and experimental results were compared for validation. The specific conclusions are as follows:

A time-varying mesh stiffness model for a spur gear pair considering single-crack and coupled-crack states was established. The stiffness when cracks appear on the driving gear and the driven gear, respectively, was compared. Based on this, the coupled influence on stiffness when both gears develop cracks simultaneously was analyzed. The stiffness under the coupled-crack condition is generally lower than when a crack appears on a single gear.

When the gear transmission system transitions from a normal state to a crack fault state, periodic impacts begin to appear in the time domain. The impact period for a single-crack state can correspond to the rotational speed of the gear with the crack fault. However, for the coupled-crack state, due to the coupled influence of cracks on both gears, adjacent impacts cannot be directly correlated with the rotational speeds of the individual gears; they need to be filtered and matched separately. The changes in the overall mean and root mean square values across the three states are not significant. Therefore, the amplitude of the periodic impacts is evaluated based on the peak value and crest factor. Quantitatively, the peak value exhibits a monotonic increase with fault severity, rising by a factor of 2 under single-crack conditions and approaching 8-fold under coupled-crack conditions relative to the normal state. In contrast, the mean and RMS values show variations below 4% across all conditions. It is concluded that the periodic impacts under the coupled-crack condition are much more pronounced than under the single-crack condition, and the fluctuation trend is more intuitive.

For a normal gear transmission system, peaks appear in the frequency domain near n*f_m_* (gear mesh frequency, *n* = 1, 2, 3…). When a single-crack fault occurs, the location of the peak frequencies remains unchanged, but the magnitude of the peaks increases, and periodic sidebands appear around them. The period of the sidebands equals the rotational frequency of the shaft where the cracked gear is located. When the fault further develops into a coupled-crack fault, the peak frequencies still remain unchanged, the magnitude of the peaks increases significantly, and simultaneously, the magnitude of the sidebands increases while their period changes. The changed period lies between the rotational frequencies corresponding to the driving gear and the driven gear.

Comparing the dynamic model of the gear transmission system with the experimentally collected signals, the waveforms in the time and frequency domains are affected by environmental noise, but the trends remain consistent. By comparing the rate of change in different time-domain indicators numerically, it is found that the error between the dynamic model solution results and the experimental results is very small; the percentage errors between simulation and experimental results for key time-domain indicators range from 0.27% to 6.74%, confirming that the prediction discrepancies can be consistently controlled within 10%. This quantitative agreement further validates the reliability of the proposed dynamic model for characterizing coupled crack fault behaviors.

Furthermore, this study holds significant engineering implications for the design of condition monitoring systems. Through revealing that peak-related indicators are more sensitive than RMS for coupled cracks, it suggests that diagnostic algorithms should prioritize impact-based features over energy-based ones to avoid missed detections. However, certain limitations exist in this work. The current study primarily focuses on spur gears under constant load conditions, and specific quantitative thresholds for fault classification were not established. Future research will address these limitations by extending the coupled fault model to helical and planetary gear systems under variable load conditions, ultimately translating these theoretical insights into practical industrial applications.

## Figures and Tables

**Table 1:** Gearing system-related parameters.

Gear Parameters	Driving Gear	Driven Gear
Equivalent modulus (mm)	6	
Tooth width (mm)	14	
Pressure angle (°)	20	
Number of teeth	16	13

**Table 2:** Time domain vibration response metrics under different conditions.

	Normal State	Single Cracked State	Rate of Change	Coupled Cracked State	Rate of Change
Max	1.3119	1.3789	105.10%	1.6906	128.86%
Min	1.2337	1.2061	97.76%	1.0495	85.06%
Mean	1.2728	1.2809	100.63%	1.3016	102.26%
RMS	1.2729	1.2812	100.65%	1.3046	102.49%
Peak	0.0782	0.1728	220.99%	0.6411	819.80%
Peak factor	0.0614	0.1349	219.56%	0.4914	799.85%

**Table 3:** Time domain vibration response metrics under different experimental groups.

	Normal State	Single Cracked State	Rate of Change	Coupled Cracked State	Rate of Change
Max	1.1090	1.1667	105.20%	1.4636	131.97%
Min	0.9807	0.8846	90.19%	0.4781	48.75%
Mean	1.0447	1.0443	99.96%	1.0095	96.63%
RMS	1.0449	1.0441	99.92%	1.0225	97.85%
Peak	0.1282	0.2820	220.00%	0.9845	767.98%
Peak factor	0.1227	0.2701	220.15%	0.9637	785.45%

**Table 4:** Percentage error between experimental and simulation results.

Percentage Error	Single Cracked State	Coupled Cracked State
Mean	0.67%	5.82%
RMS	0.72%	4.73%
Peak	0.44%	6.74%
Peak factor	0.27%	1.83%

## Data Availability

The data are available from the corresponding author on reasonable request.
